# Dysmorphic contribution of neurotransmitter and neuroendocrine system polymorphisms to subtherapeutic mood states

**DOI:** 10.1002/brb3.1140

**Published:** 2019-01-17

**Authors:** Irene Gonzalez, Rocio Polvillo, Maximiliano Ruiz‐Galdon, Armando Reyes‐Engel, Jose Luis Royo

**Affiliations:** ^1^ Department of Surgery, Biochemistry and Immunology, School of Medicine University of Malaga Malaga Spain; ^2^ Centro Andaluz de Biología del Desarrollo Seville Spain

**Keywords:** BDNF, COMT, Goldberg, HTR2A, MAO, mood state, OPRM1, POMS, SLC18A1

## Abstract

**Objective:**

From an evolutionary perspective, emotions emerged as rapid adaptive reactions that increase survival rates. Current psychobiology includes the consideration that genetic changes affecting neuroendocrine and neurotransmission pathways may also be affecting mood states. Following this hypothesis, abnormal levels of any of the aminergic neurotransmitters would be of considerable importance in the development of a pathophysiological state.

**Materials and Methods:**

A total of 668 students from the School of Medicine of the University of Malaga (Average = 22.41 ± 3; 41% men) provided self‐report measures of mood states using POMS and GHQ‐28 questionnaires and buccal cells for genotyping 19 polymorphisms from 14 selected neurotransmitter pathways genes (*HTR1A; HTR2A; HTR2C; HTR3B; TPH1; SLC18A1; SLC18A2; COMT; MAOA; MAOB*) and neuroendocrine system (*AVPR1B; OPRM1; BDNF; OXTR*).

**Results:**

*MAOA* rs3788862 genotype correlates with decreasing levels of Tension among females (beta = −0.168, *p*‐value = 0.003) but it is neutral among males in this subscale. On the contrary, it correlates with lower GHQ‐28 depression scores among males (beta = −0.196, *p*‐value = 0.008). Equivalently, *SLC18A1* and *HTR2A* variants correlated with anger and vigor scores, only among males. From the neuroendocrine system, *OPRM1* rs1799971 correlated increasing levels of female's Anxiety, depression and Social Dysfunction scores.

**Conclusion:**

Our findings suggest that these polymorphisms contribute to define general population mood levels, although exhibiting a clear sexual dimorphism.

## 1 INTRODUCTION

The study of human personality, behavior, and mood has been addressed from multiple disciplines. Understanding the intimate nature of our emotions, let us give a rational voice to the feelings that condition our behavior in society. Emotions have been explained from the evolutionary perspective as rapid adaptive reactions that increase survival rates among vertebrates (Nesse & Ellsworth, 2009). Anxiety and fear, for example, are triggered from the amygdala even before our frontal cortex processes the origin of the warning stimulus. This alarm system is tightly regulated but allows an overreaction. From a purely biological point of view, it is much more economical to be alarmed without reason than not to once a situation deserves it (Marks & Nesse, 1994, Sanjuán and Casés, 2005, Garakani, Mathew, & Charney, 2006). Certain types of depression would emerge as a strategy of energy savings once facing the impossibility of achieving an objective, therefore reducing the risk to new stressors, a situation that would be reversed when these objectives are achieved (Sanjuán and Casés, 2005; Kinney & Tanaka, 2009). In this sense, depressed patients with a poor therapeutic response exhibit a significant improvement upon facing a favorable environmental change. This is also compatible with the hypotheses related to social competition, according to which the levels of serotonin (5‐hydroxytyroxine or 5‐HT) in the central nervous system are elevated upon the achievement of dominance, which is associated with the decrease in stress levels and mood enhancement (Raleigh et al., 1991; Price, Sloman, Gardner, Gilbert, & y Rohde, 1994). Each element that contributes to the mood state is influenced by a wide spectrum of individual and collective factors; therefore, it might be considered one of the most complex human traits to study. Knowing the biochemical pathways that comprise mood states is especially relevant when associated with the daily clinical practice. This happens whenever a subject reaches a pathological level of the different components of the mood state and exhibit anxiety disorder, bipolar disorder, or major depression.

We must take into account that the different dimensions of mood states are quantitative variables that are differently affecting general population. Currently, we have a diverse scope of technical approaches to study mood states. The Goldberg General Health Questionnaire (GHQ‐28) is an instrument originally designed to identify nonpsychotic mental disorders in contexts of general medical practice. It allows to differentiate in a simple way, psychiatric patients from those considered healthy (Goldberg, 1978; Retolaza et al, 2003). The GHQ‐28 consists of four subscales: A‐scale refers to somatic symptoms, B to anxiety and insomnia, C to social dysfunction, and D to depression. GHQ‐28 can be applied to the general population and is suggested for the assessment of mental health. The Profile of Mood States (POMS) test consists of 65 items rated using a Likert type format, with five alternatives response ranging from 0 to 4 (McNair, Lorr, & Droppleman, 1971). It allows to obtain a general index of the alteration of seven partial measurements: tension, depression, anger, vigor, fatigue, confusion, and friendship. At the beginning, this test was used to evaluate the effects of psychotherapy and medication in external psychiatric patients although it was also tested with a variety of nonpsychiatric samples and has become a very popular instrument (Andrade et al., 2002).

Current psychobiology includes the consideration that genetic changes affecting neurotransmission pathways may also be affecting mood states. Following this hypothesis, abnormal levels of any of the aminergic neurotransmitters, dopamine, norepinephrine, and serotonin, would be of considerable importance in the development of a pathophysiological state (Baldwin & Birtwistle, 2002). Serotonin offers remarkable action on sleep‐wake cycle, behavior, cardiac function, endocrine secretions, pain perception, appetite, and sexual activity. Tryptophan is the known precursor of serotonin. Functional mutations affecting the coding region of the tryptophan‐hydrolase 2 gene (*TPH2*) have been found among families with bipolar disorder (Cichon et al., 2008 and Grigoroiu‐Serbanescu et al., 2008). Other studies have analyzed the role of genes involving the neurotransmitter synthesis, transport, and degradation such as *SLC6A3, HTR2A, MAOA, COMT*, and *SLC6A4* (O'Donovan et al., 2008; Williams et al., 2011). Coding variants within the *COMT* gene, related to dopamine degradation, have been shown to be associated with bipolar disorder risk (Zhang et al., 2009). A single‐nucleotide polymorphism (SNP) in the promoter region of the serotonin receptor gene *HTR1A* was also significantly associated with bipolar disorder risk (Kishi et al., 2011) as well as different genomic variants of the mono amine oxidase genes (*MAOA, MAOB*) (Fan et al., 2010). Polymorphisms within *SLC6A4* (5‐*HTTLPR*) have been studied among major depressive disorder patients and been included in several meta‐analyses that demonstrated a small but significant association to bipolar disorder (Lasky‐Su, Faraone, Glatt, & Tsuang, 2005; Cho et al., 2005). Meta‐analysis studying the different alleles of the *TPH1* gene concluded that it is not associated with major depressive disorder but rather with bipolar disorder (Halmoy et al., 2010). Other genes have been also found to affect different neuropsychiatric disorders such as the brain‐derived neurotrophic factor (*BDNF* gene), which is involved in both the pathogenesis of depression and the mechanism of action of antidepressant treatments (Duman & Monteggia, 2006; Verhagen et al., 2010). However, in spite of the role of aforementioned genes in the development of pathological status, literature is scarce about how the different genetic configurations affect mood states among healthy subjects. In order to evaluate in a quantitative manner the role of these genetic variants over the different dimensions of the mood state within the general population, we initiated a study in which 20 genetic variants affecting different neuroendocrine biochemical pathways were analyzed in a series of volunteers from the University of Malaga who phenotyped using POMS and GHQ‐28 questionnaires.

## 2 MATERIALS AND METHODS

### 2.1 DNA donors

The study subjects of this research were 668 healthy students of the University of Malaga who voluntarily decided to participate in the project. Inclusion criteria were being adult and fell healthy without apparent psychiatric disease. The following demographic variables were taken: weight, height, age, sex, and whether they were currently taking any drug treatment. DNA was extracted from buccal swap according to standard procedures. This research was carried out with the approval of the Ethics Committee of the University of Malaga and all the students signed an informed consent. This work was carried out in accordance with the principles of the Declaration of Helsinki.

### 2.2 Single‐Nucleotide Polymorphisms

Genotyping was outsourced to Genologica SL. SNP analysis was performed using the TaqMan Open Array Genotyping System from Applied Biosystems. The results obtained were processed using TaqMan Genotyper Software. The selected SNPs were a chosen from the literature among those affecting the neurotransmitter systems (*HTR1A* rs6295; *HTR2A* rs6313; *HTR2C* rs3813929; *HTR3B* rs1176744; *TPH1* rs1800532; *SLC18A1* rs1390938; rs2270641; *SLC18A2* rs363371; *COMT* rs6269, rs4633, rs4818, rs4680 *MAOA* rs3788862, rs979605; *MAOB* rs3027452) and neuroendocrine (*AVPR1B* rs28632197; *OPRM1* rs1799971; *BDNF* rs6265; *OXTR* rs2254298). Details are summarized in Supporting Information Table S1.

### 2.3 Psychological variables

Subjects completed two online tests: the Goldberg general health questionnaire (GHQ‐28) and the Profile of Mood State (POMS) test, basing their responses on their mood status along the past few weeks. GHQ‐28 is a self‐administered questionnaire of 28 items divided into four subscales: A (somatic symptoms), B (anxiety and insomnia), C (social dysfunction), and D (Depression) (Goldberg, 1978; Andrade et al., 2002; Retolaza et al., 2003). GHQ‐28 stablishes two different scores for each subscales: new onset and chronic symptoms, depending on the internal punctuation of the different items. Along the same session, volunteers also completed the Spanish version of the POMS questionnaire, composed of 48 items, referred to six affective states: tension, depression, anger, vigor, fatigue, and friendship. For each variable, a T‐Score is computed, as a standardization of the score obtained in each item depending on the standard deviation and the mean. Total Mood Disturbance (TMD) is calculated from the T‐scores by summing negative moods minus positive emotional responses (McNair, D. M., 1992; Andrade‐Fernandez EM, 2002).

### 2.4 Statistical analysis

Hardy–Weinberg equilibrium was assayed using the corresponding online web tool from the Institute of Human Genetics of Munich (https://ihg.gsf.de/ihg/snps.html) (RRID: https://scicrunch.org/resolver/SCR_016496). Statistical analysis was performed using IBM SPSS Statistics v22 (RRID: https://scicrunch.org/resolver/SCR_002865). Graphical representations were generated both with the IBM SPSS program and with the Microsoft Excel spreadsheet. Kolmogorov–Smirnov test was used to determine the normality of the quantitative data series. For bivariate correlations studies, both the Pearson's correlation coefficient and Spearman's Rho were calculated. For models that included both the genetic variants and other covariates, the linear regression models were used. The level of significance was 0.05.

## 3 RESULTS

The study comprised 668 students from the School of Medicine of the University of Malaga recruited between 2011 and 2015. The age of the study subjects was relatively homogeneous (22.41 ± 3 years) although ranged between 18 and 51 years. The series was composed by 41% men and all from Caucasian origin. All of them were sampled for buccal swap for subsequent determination of genetic polymorphisms. Call ratios had an average of 96%, although they ranged from 98% for SNPs such as rs3813929, rs3027452, or rs2254298, and the minimum of 89% obtained with rs6313. Hardy–Weinberg equilibrium (HWE) was determined for those SNPs mapping autosomal chromosomes and only those with *p* > 0.05 were used for further analyses (all but rs2254298, rs324981, and rs1800532, Supporting Information Table S2). When volunteers were then invited to fill the POMS and GHQ‐28 questionnaires, from the initial 668 students, 601 (90%) completed both tests. Regarding the variables under study, a summary of the mood variables determined using POMS and GHQ‐28 is shown in Supporting Information Table S3.

We first determined the correlation between both tests and evaluated the effects attributed to age, sex, or BMI (Supporting Information Table S4). Gender exhibited statistically significant differences in Vigor T‐score (lower among females, Spearman's *p*‐value = 0.004) and chronic Anxiety (higher among females, Spearman's *p*‐value = 0.008). Age also correlated with different parameters such as vigor, friendship, and new onset Depression, evidencing the need to use them as covariates to determine the potential role of the genetic variants under analyses. Beyond this, we found a relevant intercorrelation between the different variables within the same questionnaire (GHQ‐28 chronic and new onset) as well as a significant correlation between equivalent variables interrogated in POMS and GHQ‐28. As an example, we found that the POMS T‐score measuring fatigue positively correlated with GHQ‐28 chronic anxiety and depression levels (Rho > 0.435, *p*‐value < 0.001) (Supporting Information Table S4). Therefore, both test might be considered to a certain extend an internal replica.

**Figure 1:**
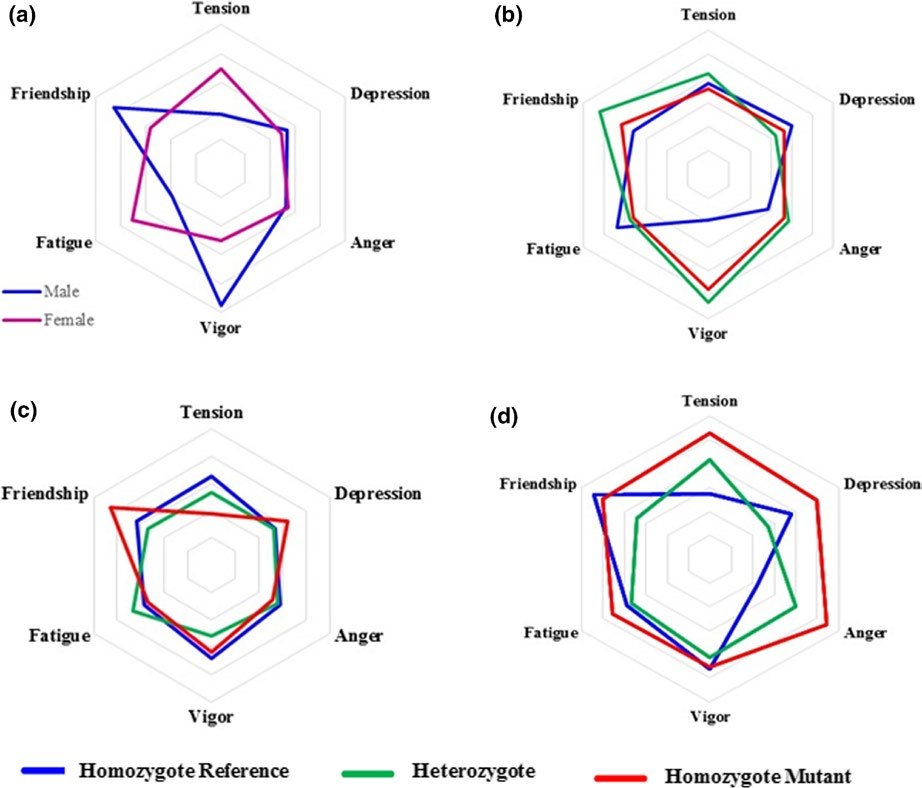
Representative Spider diagrams of the scores obtained from the POMS questionnaires. Panel a represents the average score for each subscale of the POMS questionnaire according to gender. Panel b represents the average scores for each of the three *HTR2A* rs6313 genotype. Panel c refers to *MAOA* rs3788862, and Panel d to *SLC18A1* rs2270641

Next, we performed a multiple correlation analysis between the three genotypes for each genetic variant and the POMS T‐scores. Results are shown in Table 1. A particular haplotype captured by the two variants within the *MAOA* gene correlated with a lower degree of Tension. *HTR2A* rs6313 also correlated with Vigor (Rho = 0.134, *p*‐value = 0.004) suggesting that those subjects harboring the mutant homozygous genotype reported an increased Vigor than those with the reference genotype. We might mention the associations found for *SLC18A1* variants and *BDNF* rs6265; however, the *p*‐values obtained do not support multiple correction and therefore should be treated with caution. Of mention, none of the variants analyzed correlated with the Total Mood Disturbance T‐score, pointing that the individual effects of any of the genetic variants under study are not enough per se to generate a significant impact over the general mood state of a subject. We should also highlight that we have assayed a codominant genetic model of inheritance assayed. This can be graphically visualized using Spider diagrams where in a codominant genetic model the effect of the heterozygote genotype is between the two homozygotes (Figure 1). When the genetic variants were correlated with the different subscales assessed by the GHQ‐28 test, we found some discrepancies depending on whether they were constructed, this is, new onset versus the chronic subscale (Table 2). Variants mapping the *COMT* gene (rs4680, rs6269, and rs4818) correlated with Anxiety of new onset, but not when defined as a chronic variable. Similar results were obtained for *HTR2A* rs6313 and *MAOA* rs3788862 for anxiety and depression. In fact, we found a higher proportion of significant associations within the new onset construct, suggesting that this test might be especially sensitive to mood disturbances. We must however highlight the effect of the *OPRM1* variant rs1799971, which was consistently associated, in both constructs, with Anxiety (Rho > 0.099, *p*‐value < 0.028). Moreover, this variant correlated with less Social Dysfunction (Rho = −0.176, *p*‐value < 0.001) and higher somatic scores (Rho = 0.114, *p*‐value = 0.011).

**Table 1:** Association between the genetic variants and the POMS variables measured

Gene	SNP	T‐SCORES													
		Tension		Depression		Cholera		Vigor		Fatigue		Friendship		TMD	
		Rho	*p*‐value	Rho	*p*‐value	Rho	*p*‐value	Rho	*p*‐value	Rho	*p*‐value	Rho	*p*‐value	Rho	*p*‐value
BDNF	rs6265	−0.033	0.462	−0.062	0.167	−0.036	0.422	0.025	0.583	0.003	0.953	**0.094**	**0.038**	−0.017	0.701
COMT	rs4680	0.019	0.672	−0.051	0.257	−0.019	0.673	−0.009	0.841	0.006	0.896	0.014	0.755	0.009	0.843
	rs6269	0.006	0.897	0.053	0.241	0.031	0.496	0.008	0.866	0.015	0.745	−0.036	0.420	0.006	0.901
	rs4633	0.021	0.636	−0.051	0.256	−0.033	0.459	−0.007	0.875	0.010	0.824	0.029	0.517	0.011	0.800
	rs4818	−0.003	0.952	0.046	0.312	0.027	0.546	0.017	0.707	−0.012	0.794	−0.035	0.432	−0.006	0.902
HTR1A	rs6295	0.034	0.496	0.037	0.451	0.029	0.559	−0.003	0.947	0.047	0.342	−0.017	0.730	0.045	0.365
HTR2A	rs6313	−0.013	0.782	−0.030	0.524	0.053	0.259	**0.134**	**0.004**	−0.012	0.796	0.047	0.319	−0.033	0.488
HTR2C	rs3813929	0.072	0.109	0.020	0.655	0.008	0.856	−0.042	0.352	0.075	0.095	0.024	0.595	0.066	0.145
HTR3B	rs1176744	0.007	0.872	−0.011	0.807	−0.044	0.335	0.011	0.811	−0.021	0.640	0.026	0.571	−0.022	0.635
MAOA	rs3788862	**−0.098**	**0.029**	0.011	0.804	−0.008	0.858	−0.053	0.240	0.014	0.748	0.024	0.590	−0.002	0.962
	rs979605	**−0.136**	**0.003**	−0.013	0.776	−0.008	0.859	−0.050	0.271	−0.013	0.782	0.003	0.945	−0.035	0.440
MAOB	rs3027452	0.042	0.355	0.024	0.597	0.047	0.300	−0.028	0.535	0.051	0.257	−0.023	0.612	0.050	0.268
OPRM1	rs1799971	0.049	0.278	0.038	0.395	0.071	0.117	−0.020	0.661	0.049	0.278	−0.012	0.787	0.047	0.303
SLC18A1	rs2270641	0.069	0.127	−0.014	0.757	**0.090**	**0.047**	0.008	0.858	0.002	0.965	−0.041	0.362	0.028	0.538
	rs1390938	−0.039	0.392	−0.022	0.626	−0.038	0.396	**−0.091**	**0.044**	−0.041	0.362	−0.053	0.242	−0.028	0.531
SLC18A2	rs363371	−0.019	0.672	−0.085	0.059	−0.002	0.958	0.028	0.543	0.023	0.619	−0.083	0.066	−0.044	0.335

Note

Statistically significant values are highlighted in bold.

**Table 2:** Association between the genetic variants and the GHQ‐28 subscales measured

Gene	SNP	New onset							
		A (Somatization)		B (Anxiety)		C (Social Dysfunction)		D (Depression)	
		Rho	*p*‐value	Rho	*p*‐value	Rho	*p*‐value	Rho	*p*‐value
BDNF	rs6265	−0.017	0.704	0.001	0.988	0.013	0.782	0.020	0.666
COMT	rs4680	−0.050	0.263	**−0.092**	**0.042**	0.088	0.051	−0.011	0.810
	rs6269	0.074	0.102	**0.094**	**0.037**	−0.060	0.186	−0.013	0.775
	rs4633	−0.045	0.320	−0.086	0.057	0.088	0.052	−0.014	0.755
	rs4818	0.064	0.156	**0.107**	**0.017**	−0.065	0.148	0.013	0.767
HTR1A	rs6295	−0.015	0.756	−0.019	0.697	0.010	0.846	0.002	0.961
HTR2A	rs6313	**0.109**	**0.020**	**0.107**	**0.022**	−0.088	0.059	0.021	0.657
HTR2C	rs3813929	−0.036	0.424	−0.014	0.763	0.018	0.696	−0.035	0.436
HTR3B	rs1176744	0.002	0.967	−0.072	0.112	0.036	0.425	−0.075	0.097
MAOA	rs3788862	−0.039	0.384	**−0.090**	**0.046**	0.073	0.106	**−0.095**	**0.035**
	rs979605	−0.074	0.102	−0.060	0.189	0.022	0.629	−0.046	0.309
MAOB	rs3027452	−0.058	0.200	−0.048	0.284	−0.043	0.341	−0.035	0.437
OPRM1	rs1799971	**0.114**	**0.011**	**0.099**	**0.028**	**−0.176**	**0.000**	0.051	0.261
SLC18A1	rs2270641	−0.024	0.599	−0.012	0.799	−0.041	0.360	0.031	0.499
	rs1390938	−0.013	0.769	−0.041	0.365	0.023	0.605	−0.013	0.775
SLC18A2	rs363371	−0.042	0.358	−0.015	0.734	0.005	0.918	−0.057	0.208

Gene	SNP	Chronic							
		A (Somatization)		B (Anxiety)		C (Social Dysfunction)		D (Depression)	
		Rho	*p*‐value	Rho	*p*‐value	Rho	*p*‐value	Rho	*p*‐value
BDNF	rs6265	−0.035	0.440	−0.038	0.404	−0.002	0.961	−0.039	0.386
COMT	rs4680	−0.005	0.915	−0.031	0.493	−0.008	0.866	0.023	0.606
	rs6269	0.013	0.774	0.011	0.804	−0.009	0.849	−0.041	0.364
	rs4633	−0.009	0.851	−0.034	0.451	−0.001	0.982	0.023	0.611
	rs4818	−0.010	0.826	−0.006	0.899	−0.021	0.645	−0.020	0.660
HTR1A	rs6295	−0.059	0.232	0.042	0.398	−0.006	0.910	0.051	0.300
HTR2A	rs6313	0.039	0.401	−0.002	0.973	0.026	0.583	0.043	0.362
HTR2C	rs3813929	0.005	0.912	0.013	0.778	0.024	0.595	−0.010	0.818
HTR3B	rs1176744	−0.057	0.211	−0.073	0.108	0.008	0.862	−0.013	0.774
MAOA	rs3788862	0.032	0.483	0.021	0.643	0.013	0.779	0.025	0.574
	rs979605	0.051	0.260	−0.012	0.784	0.011	0.802	0.009	0.844
MAOB	rs3027452	0.058	0.200	0.074	0.100	0.085	0.059	0.036	0.430
OPRM1	rs1799971	0.081	0.074	**0.122**	**0.007**	0.029	0.526	−0.002	0.966
SLC18A1	rs2270641	−0.023	0.615	−0.011	0.807	−0.007	0.878	0.043	0.342
	rs1390938	0.051	0.263	−0.006	0.894	0.040	0.372	0.017	0.712
SLC18A2	rs363371	0.006	0.903	−0.012	0.798	0.064	0.157	−0.019	0.672

Note

Statistically significant values are highlighted in bold.

Finally, we performed a regression analysis adjusted by age and stratified by sex in order to subtract the effect of these variables and quantify the net effect of the genetic variants. Results recapitulate to a large extend the associations captured in the univariate models; however, we identified a clear sexual dimorphism for several genetic variants (Table 3). Regarding the neurotransmitter system selected genes, we can highlight the role of *MAOA*, whose mutant alleles correlate with decreasing levels of tension (within the POMS questionnaire) among females but it was neutral among males for this subscale. On the contrary, rs3788862 seemed to correlate with less Depression levels among males (beta = −0.196, *p*‐value = 0.008) when quantified with GHQ‐28 while being neutral among females. Equivalently, *SLC18A1* and *HTR2A* variants correlated with increasing levels of anger and vigor, respectively, but only among males. From the neuroendocrine system‐associated genes, we might highlight the association among females between *OPRM1* polymorphism and increasing levels of anxiety and somatization, concomitantly with lower Social Dysfunction scores.

**Table 3:** Association between the genetic variants and the POMS and GHQ‐28 dimensions measured stratified by sex and adjusted by age

Gene	SNP	T‐score	POMS					
			Male			Female		
			Beta	T	*p*‐value	Beta	T	*p*‐value
HTR2A	rs6313	Vigor	0.207	2.727	**0.007**	0.060	1.006	0.315
BDNF	rs6265	Friendship	−0.001	−0.013	0.990	0.157	2.751	**0.006**
MAOA	rs3788862	Tension	−0.011	−0.146	0.884	−0.168	−2.966	**0.003**
	rs979605	Tension	−0.076	−1.013	0.312	−0.162	−2.830	**0.005**
SLC18A1	rs2270641	Anger	0.184	2.497	**0.013**	0.090	1.564	0.119

Gene	SNP	New Onset	GHQ‐28					
			Male			Female		
			Beta	T	*p*‐value	Beta	T	*p*‐value
HTR2A	rs6313	A (Somatization)	0.037	0.475	0.636	0.141	2.394	**0.017**
OPRM1	rs1799971		0.093	1.275	0.204	0.128	2.242	**0.026**
		B (Anxiety)	0.053	0.729	0.467	0.138	2.416	**0.016**
		C (Social Dis.)	−0.075	−1.023	0.308	−0.185	−3.271	**0.001**
MAOA	rs3788862	D (Depression)	−0.196	−2.698	**0.008**	−0.036	−0.621	0.535

Gene	SNP	Chronic	Male			Female		
			Beta	T	*p*‐value	Beta	T	*p*‐value
OPRM1	rs1799971	B (Anxiety)	−0.001	−0.014	0.989	0.177	3.116	**0.002**

Note

Statistically significant values are highlighted in bold.

## 4 DISCUSSION

The present study shows the quantitative evaluation of functionally relevant variants of the neuroendocrine and neurotransmitter systems of the mood state of a cohort representative from the general population. We have characterized the correlation between 16 selected SNPs and the different items included in two widely used and validated psychometric mood questionnaires. The obtained results show a significant correlation between equivalent items of each test. More importantly, we found statistically significant associations between different items with and the subject's genotype. There are three worth noting aspects, first, that our study is based on a young and healthy population where the different mood subscales show a subtherapeutic continuum, in contrast to most of the works reporting differences between cases and controls of a particular pathology. Secondly, we should highlight the clear sexual dysmorphic mood response and genetic correlation, suggesting that the genetic background differentially compromises the mood state depending on the gender. And third, the degree of coherence obtained in the results between the functional attributions of each selected polymorphism and the emotional items to which they have been associated. It is necessary to keep in mind that when a study of genetic association is made, the phenotype must be sufficiently marked to exhibit a statistically significance. In our case, the population phenotypes should be considered as euthymic, what means that the results obtained detect genetic associations with emotional conditions that did not give rise to a psychopathology. This sensitivity on detection mood variability and genotype among euthymic subjects could be supported by the high homogeneity of the age a sociocultural features of the population studied. Perhaps because of this, some statistical association may not support multiple testing correction.

Similar studies (Takeuchi et al., 2015) relate the polymorphism of *DRD2* with the POMS test and find differences between sexes in a similar population. Yarosh, Meda, Wit, Hart, & Pearlson, (2015), performed a multivariate analysis of polymorphisms of a whole genome association study with the POMS test in healthy subjects treated with amphetamine, finding association with SNPs related to genes of the glutamatergic signal pathways, which seem to mediate behavior and in cardiovascular responses to amphetamine.

On the other hand, in the present study a sexual dimorphism is shown both when we correlate general items such as age, sex, and BMI and when we observe them associated with genotypes. Among the general items such as BMI, women have lower BMI than men in our population, which is reversed in the adult population, perhaps due to the low average age of the sample (Wells, 2007). The male sex correlates positively with the vigor. Age correlates positively with BMI (Livshits et al., 2012), and negatively with anger. This points that age correlates with lesser anger, higher vigor, friendship, and somatization. Thus, the irritable attitude decreases with age, as the sensation of activity and energy increases, what taking into account the age range of the population this could be associated with a greater hormonal balance, increases the capacity of relationships that is translated into friendship, and somatic sensations increase maybe due to greater recognition of one's body.

The correlations between the test items show that tension, nervousness, agitation, etc., positively correlate with depression, anger, fatigue, and TMD of POMS questionnaire and anxiety, somatization, depression, and social dystonia in their chronic profile from GHQ‐28 and negatively with the vigor and friendship (POMS questionnaire). This stress pattern is the same for depression, anger, fatigue and TMD and chronic GHQ‐28. On the contrary, friendship and vigor behave inversely, give results of direct correlation with themselves and inverse with all others. It is evident that the nervous alteration is directly related to all the states that are proposed of imbalance and inversely with the states that define more balance, friendship, and vigor. It is noteworthy that the new onset of the GHQ‐28 test items does not correlate with the other items, perhaps due to some general premise about this test that nullifies the possibility of correlation, since this absence of significance is very strange.

When splitting the population by gender, we observe a strong dimorphic and excluding distribution, that is, associations occur only in one sex and never in the other. Thus, vigor is associated only to the male sex by *HTR2A*,* BDNF* to friendship only in women, *MAOA* to a lesser tension score only among women and *SLC18A1* to anger only within men (all from the POMS test). The same dimorphic and excluding pattern occurs when GHQ‐28 is assayed: *HTR2A* and *OPRM1* for somatization only among women, *OPRM1* for new onset and chronic anxiety among women. *MAOA* genotype behaves as an antidepressant only among males. In order to stablish a functional relationship for these results, we found that our results show a relation of the alleles classified as lower MAO activity with the lesser tension in woman measured by the POMS test and with the Anxiety, in woman, and no depression, with the GHQ‐28 test in man. These results are consistent with those found previously on their association with depression and with the well‐known effect of MAO inhibitors (MAOI) on depressive status (Lung, Tzeng, Huang, & Lee, 2011). Another previous study on *MAO* polymorphisms found an association with negative mood especially with *MAOB* and *MAOA* haplotypes. In this test, subjects are classified by negative or positive emotional attitudes, finding a relation with *MAOB* polymorphism with negative mood (Dlugos, Palmer, & Wit, 2009). However, we fail to replicate these results for *MAOB* rs3027452. Since these genes map the X chromosome, the sexual dimorphism found for these genes can be considered as a dose‐dependent effect. Thus, women shall be more predisposed to depression and less tension, inversely than men, according to a higher MAO activity found among women (Jansson, 2005).

Regarding the neuroendocrine system genes, we should highlight that *BDNF* rs6265 encodes for a Valine (Val) 66 to Methionine (Met) change. The most common allele (G) encodes for Val while the mutant allele (A) encodes Met. Statistically significant differences were found between the Val/Met genotypes and the response to emotional expressions measured by functional magnetic resonance imaging signals in the orbitofrontal cortex, amygdala, and hippocampus on the passive view of faces with different expressions (Gasic et al., 2009). *BDNF* genotypes represented approximately 30% of the variance of reward/aversion reactions demonstrating that these allelic variants clearly influence the interpretation of reality. Our results show a relationship of the Val allele with a higher degree of sociability only among women, perhaps related to the emotional control of this same circuit. *OPRM1* rs1799971 also correspond to a coding variant. In this case, mapping exon 1 of the mu opioid receptor and causing that the normal amino acid at residue 40, asparagine (Asn), to be replaced by aspartic acid (Asp). According to the literature, mutant allele is related to increased pain, suggesting a compromised protein function (Slavich, 2014). In our study, we found an association to increased anxiety and somatization symptoms, while decreases Social Dysfunction, but only among women. This, in terms of quality of life, could be substantiated in a greater sensitivity to pain and less pleasant rewards to intense stimuli. Variant G carriers are adapted to relaxation stimuli or lower endorphinic pleasure than carriers of A. In fact could explain why this polymorphism and in particular the G allele is associated with a greater tendency to addiction and variations in the pharmacological response to it (van den Wildenberg et al., 2007; Anton et al., 2008). We did not find in the literature any association study on emotional response, most of them refer to predisposition to addiction, and some to depression, but indirect processes as a consequence of pain. Our results show an association in the responses to the Goldberg test of the G allele with anxiety and insomnia, somatization and very strongly negatively associated with social dysfunction among women. This result indicates that subjects in whom the degree of neurological reward mediated by the opioid effect is diminished are the most socially adapted or those with less social dysfunction indicate always that not fall in addiction. It could be interpreted that their state of less neurological pleasure leads to a more correct social response, perhaps abounding on the hypothesis that the greater tendency to pleasure is associated with a greater rebellion to social restrictions (Slavich, Tartter, Brennan, & Hammen, 2014). Overall, it can be deduced that genetic variations within the neurotransmitter (HTR2A, SLC18A1, MAOA) and neuroendocrine (BDNF and OPRM1) systems, determined in a euthymic population, are associated with emotional traits quantitatively assays using POMS and GHQ‐28 questionnaires, although the gender shall be considered as both determining and excluding criteria in the different associations.

### 4.1 Study limitations

Whenever a genetic association is reported, the suspect of facing a false positive arises. Given the amount of variables assayed, we might consider that defining an alpha of 0.05 might be too permissive. Here, we analyzed 14 genes and conducted two mood tests, but the different variables under study are not purely independent. MAOA polymorphisms for instance are in linkage disequilibrium. In the same line, several dimensions measured with Goldberg's and GHQ‐28 questionnaires show a statistical correlation. Therefore, some corrections such as Bonferroni adjustment shall be considered too exhaustive. A second limitation of the study relates to the genetic heterogeneity of the Spanish South Eastern population. This would eventually have included a microestratification effect. However, we should highlight the population was composed of volunteers with Caucasian and that the Spanish population is largely homogeneous (Gayan et al., 2010). For these reasons, an independent replica of the current findings would be required to confirm our findings.

## CONFLICT OF INTERESTS

The authors declared no potential conflicts of interest with respect to the research, authorship, and/or publication of this article. This work was financed using internal sources from the Department of Surgery, Biochemistry and Immunology, Universidad de Málaga.
