# Supplementary material for: Dysmorphic contribution of neurotransmitter and neuroendocrine system polymorphisms to subtherapeutic mood states
Source: Brain Behav. 2019 Jan 17;9(2):e01140. doi: 10.1002/brb3.1140 (PMC6379594; doi:10.1002/brb3.1140)
Supplement: Supplementary file 1 [file BRB3-9-e01140-s001.docx]

**Table S1**. Polymorphisms analyzed in the study.

| Gene | SNP ID | Cr. | Position hg18 | SNP | Reference allele | Description |
| --- | --- | --- | --- | --- | --- | --- |
| AVPR1B | rs28632197 | 1 | 204397331 | C/T | T | Arginine Vasopressin Receptor 1B. Related to oxytocin receptors. |
| OXTR | rs2254298 | 3 | 8760542 | A/G | G | Oxitocin receptor. |
| HTR1A | rs6295 | 5 | 63962738 | C/G | C | 5-hydroxytryptamine receptor 1. Monoamine receptor |
| OPRM1 | rs1799971 | 6 | 154039662 | A/G | A | Mu Opioid receptor. Target of endogenous opioid peptides |
| SLC18A1 | rs1390938 | 8 | 20179202 | A/G | A | Solute carrier family 18 vesicular monoamine 1 and Solute carrier family 18 vesicular monoamine 2. Monoamine transporters |
|  | rs2270641 | 8 | 20180955 | G/T | T |  |
| SLC18A2 | rs363371 | 10 | 117226885 | A/G | G |  |
| TPH1 | rs1800532 | 11 | 18026269 | A/C | C | Tryptophan hydroxylase 1. Involved in Monoamine biosynthesis. |
| BDNF | rs6265 | 11 | 27658369 | A/G | G | Brain Derived Neurotrophic Factor. Related to synaptic plasticity and intrinsic neuronal excitability |
| HTR3B | rs1176744 | 11 | 113932306 | G/T | G | 5-hydroxytryptamine receptor 3B.  Monoamine receptors |
| HTR2A | rs6313 | 13 | 46895805 | C/T | C | 5-hydroxytryptamine receptor 1. Monoamine receptors |
| COMT | rs6269 | 22 | 19962429 | A/G | G | Catechol-O-methyltransferase. Protein involved in Monoamine degradation |
|  | rs4633 | 22 | 19962712 | C/T | C |  |
|  | rs4818 | 22 | 19963684 | C/G | G |  |
|  | rs4680 | 22 | 19963748 | A/G | G |  |
| MAOA | rs3788862 | X | 43658116 | A/G | A | Monoamine Oxidases A and B.  Involved in Monoamine degradation |
|  | rs979605 | X | 43742116 | C/T | T |  |
| MAOB | rs3027452 | X | 43798542 | A/G | G |  |
| HTR2C | rs3813929 | X | 114584047 | C/T | C | Hydroxytryptamine receptor 2C. Monoamine receptor. |

**Table S2**. Genotype calls and Hardy-Weinberg Equilibrium.

| **Gene/SNP** | **AVPR1B** | **OXTR** | **HTR1A** | | **OPRM1** | **SLC18A1** | | | | **SLC18A2** | **TPH1** | | **BDNF** | | **HTR3B** |
| --- | --- | --- | --- | --- | --- | --- | --- | --- | --- | --- | --- | --- | --- | --- | --- |
|  | rs28632197 | rs2254298 | rs6295 | | rs1799971 | rs1390938 | | | rs2270641 | rs363371 | rs1800532 | | rs6265 | | rs1176744 |
| **Homo Ref** | 521 | 466 | 167 | | 428 | 406 | | | 261 | 436 | 245 | | 383 | | 272 |
| **Het** | 120 | 160 | 289 | | 191 | 218 | | | 287 | 191 | 284 | | 233 | | 286 |
| **Homo mt** | 1 | 26 | 88 | | 31 | 22 | | | 94 | 24 | 120 | | 31 | | 81 |
| **MAF** | 0.10 | 0.16 | 0.43 | | 0.19 | 0.20 | | | 0.37 | 0.18 | 0.40 | | 0.23 | | 0.35 |
| **F** | 0.08 | 0.10 | -0.09 | | 0.06 | -0.04 | | | 0.04 | 0.02 | 0.09 | | -0.02 | | 0.02 |
| **HWE p-value** | 0.02 | 0.01 | 0.05 | | 0.13 | 0.33 | | | 0.31 | 0.60 | 0.02 | | 0.65 | | 0.66 |
| **Gene/SNP** | **HTR2A** | | **COMT** | | | | | | | **MAOA** | | | **MAOB** | **HTR2C** | |
|  | rs6313 | | rs6269 | rs4633 | | | rs4818 | rs4680 | | rs3788862 | | rs979605 | rs3027452 | rs3813929 | |
| **Homo Ref** | 180 | | 205 | 204 | | | 219 | 208 | | 395 | | 395 | 526 | 494 | |
| **Het** | 317 | | 315 | 308 | | | 304 | 306 | | 158 | | 137 | 114 | 106 | |
| **Homo mt** | 100 | | 133 | 140 | | | 128 | 139 | | 101 | | 109 | 17 | 56 | |
| **MAF** | 0.43 | | 0.44 | 0.45 | | | 0.43 | 0.45 | | 0.28 | | 0.28 | 0.11 | 0.17 | |
| **F** | -0.08 | | 0.02 | 0.05 | | | 0.05 | 0.05 | | NA | | NA | NA | NA | |
| **HWE p-value** | 0.06 | | 0.58 | 0.24 | | | 0.23 | 0.18 | | NA | | NA | NA | NA | |

Legend: Homo Ref stands for Homozygous for the reference allele, Het stands for heterozygous and homo mt, for homozygous for the mutant allele. MAF stads for Minor Alelle frequency. F in the imbreeding coefficient. NA stands for Not available.

**Table S3**. Description of the variables measured

| **TEST** | **Variable** | | **Average** | **Min** | **Max** | **Median** | **SD** | **Curtosis** | **Asimetry** |
| --- | --- | --- | --- | --- | --- | --- | --- | --- | --- |
| POMS | T-Score | Tension | 49.95 | 32.22 | 78.17 | 49.45 | 9.96 | -0.49 | 0.41 |
|  |  | Depression | 49.95 | 39.08 | 92.57 | 47.99 | 9.96 | 2.48 | 1.48 |
|  |  | Anger | 50.00 | 37.74 | 86.53 | 48.75 | 10.01 | 1.15 | 1.11 |
|  |  | Vigor | 50.03 | 14.63 | 69.77 | 51.39 | 9.95 | -0.01 | -0.43 |
|  |  | Fatigue | 49.95 | 34.17 | 78.61 | 48.98 | 9.95 | -0.31 | 0.53 |
|  |  | Friendship | 50.05 | 18.48 | 70.63 | 49.77 | 9.93 | 0.08 | -0.24 |
|  |  | TMD | 199.87 | 128.23 | 339.21 | 194.82 | 35.13 | 0.23 | 0.70 |
| GHQ-28 | Chronic | A (Somatization) | 3.18 | 0.00 | 8.00 | 3.00 | 2.15 | -0.65 | 0.45 |
|  |  | B (Anxiety) | 2.66 | 0.00 | 6.00 | 2.00 | 1.87 | -1.13 | 0.17 |
|  |  | C (Social Dysf.) | 1.80 | 0.00 | 7.00 | 1.00 | 1.45 | 2.35 | 1.71 |
|  |  | D (Depression) | 0.89 | 0.00 | 7.00 | 0.00 | 1.50 | 5.50 | 2.31 |
|  | New Onset | A (Somatization) | 5.97 | 0.00 | 8.00 | 7.00 | 1.75 | 1.88 | -1.54 |
|  |  | B (Anxiety) | 4.95 | 0.00 | 6.00 | 6.00 | 1.52 | 1.50 | -1.52 |
|  |  | C (Social Dysf.) | 1.65 | 0.00 | 6.00 | 1.00 | 1.28 | 2.26 | 1.66 |
|  |  | D (Depression) | 6.72 | 0.00 | 7.00 | 7.00 | 0.89 | 26.16 | -4.73 |

**Table** **S4**. Intercorrelation between the subscales measured with POMS and GHQ-28

|  | | | | **POMS** | | | | | | | **GHQ-28** | | | | | | | |
| --- | --- | --- | --- | --- | --- | --- | --- | --- | --- | --- | --- | --- | --- | --- | --- | --- | --- | --- |
|  |  |  |  | **T-Score scales** | | | | | | | **Chronic** | | | | **New apearance** | | | |
|  |  |  |  | Tension | Depression | Anger | Vigor | Fatigue | Friendship | TMD | A (Somatization) | B (Anxiety) | C (Social Dosf.) | D (Depression) | A (Somatization) | B (Anxiety) | C (Social Dis.f.) | D (Depression) |
|  | | Gender | Rho | .056 | -.031 | .003 | **-.116** | .068 | -.068 | .037 | -.029 | **.109** | -.002 | .063 | -.011 | -.006 | .077 | -.040 |
|  |  |  | p | .169 | .449 | .943 | **.004** | .098 | .096 | .364 | .524 | **.008** | .970 | .125 | .779 | .883 | .061 | .332 |
|  |  | BMI | Rho | -.042 | .063 | .030 | -.043 | .018 | .012 | .028 | -.022 | -.029 | .075 | .071 | -.043 | -.028 | -.025 | -.038 |
|  |  |  | p | .307 | .124 | .462 | .296 | .656 | .776 | .501 | .630 | .476 | .068 | .083 | .300 | .489 | .542 | .350 |
|  |  | Age | Rho | .080 | -.068 | **-.081** | **.098** | -.002 | **.085** | -.009 | **.112** | .032 | -.004 | -.040 | .069 | .015 | -.010 | **.087** |
|  |  |  | p | .050 | .094 | **.048** | **.016** | .967 | **.037** | .823 | **.013** | .432 | .916 | .331 | .090 | .722 | .800 | **.034** |
| **POMS** | **T-Scores** | Tension | Rho |  | **.473** | **.564** | **-.178** | **.465** | **-.120** | **.735** | **.227** | **.651** | **.265** | **.384** | .022 | -.010 | -.013 | .047 |
|  |  |  | p |  | **.000** | **.000** | **.000** | **.000** | **.003** | **.000** | **.000** | **.000** | **.000** | **.000** | .592 | .810 | .752 | .251 |
|  |  | Depression | Rho |  |  | **.596** | **-.453** | **.555** | **-.203** | **.786** | **.117** | **.505** | **.460** | **.547** | .007 | -.058 | -.005 | -.007 |
|  |  |  | p |  |  | **.000** | **.000** | **.000** | **.000** | **.000** | **.009** | **.000** | **.000** | **.000** | .864 | .154 | .901 | .873 |
|  |  | Anger | Rho |  |  |  | **-.270** | **.413** | **-.287** | **.709** | **.159** | **.561** | **.308** | **.394** | -.010 | -.021 | -.017 | .011 |
|  |  |  | p |  |  |  | **.000** | **.000** | **.000** | **.000** | **.000** | **.000** | **.000** | **.000** | .809 | .608 | .676 | .780 |
|  |  | Vigor | Rho |  |  |  |  | **-.400** | **.419** | **-.520** | **-.076** | **-.322** | **-.419** | **-.313** | -.041 | .009 | -.017 | -.019 |
|  |  |  | p |  |  |  |  | **.000** | **.000** | **.000** | **.089** | **.000** | **.000** | **.000** | .319 | .834 | .680 | .648 |
|  |  | Fatigue | Rho |  |  |  |  |  | **-.151** | **.767** | **.170** | **.435** | **.329** | **.348** | .022 | -.003 | -.044 | .003 |
|  |  |  | p |  |  |  |  |  | **.000** | **.000** | **.000** | **.000** | **.000** | **.000** | .586 | .950 | .277 | .934 |
|  |  | Friendship | Rho |  |  |  |  |  |  | -.053 | -.087 | **-.193** | **-.198** | **-.130** | .015 | .068 | -.035 | .014 |
|  |  |  | p |  |  |  |  |  |  | .191 | .052 | **.000** | **.000** | **.001** | .719 | .095 | .393 | .732 |
|  |  | TMD | Rho |  |  |  |  |  |  |  | **.190** | **.651** | **.435** | **.526** | .035 | -.011 | -.037 | .027 |
|  |  |  | p |  |  |  |  |  |  |  | **.000** | **.000** | **.000** | **.000** | .395 | .796 | .367 | .506 |
| **Goldberg** | **Chronic** | A  (Somatization) | Rho | **.227** | **.117** | **.159** | -.076 | **.170** | -.087 | **.190** |  | **.262** | **.102** | **.138** | .087 | .029 | -.026 | .000 |
|  |  |  | p | **.000** | **.009** | **.000** | .089 | **.000** | .052 | **.000** |  | **.000** | **.023** | **.002** | .053 | .520 | .561 | .993 |
|  |  | B  (Anxiety) | Rho | **.651** | **.505** | **.561** | **-.322** | **.435** | **-.193** | **.651** |  |  | **.411** | **.482** | .023 | .000 | .015 | -.047 |
|  |  |  | p | **.000** | **.000** | **.000** | **.000** | **.000** | **.000** | **.000** |  |  | **.000** | **.000** | .582 | .996 | .718 | .247 |
|  |  | C  (Social Disf.) | Rho | **.265** | **.460** | **.308** | **-.419** | **.329** | **-.198** | **.435** |  |  |  | **.380** | .019 | -.034 | .023 | -.033 |
|  |  |  | p | **.000** | **.000** | **.000** | **.000** | **.000** | **.000** | **.000** |  |  |  | **.000** | .649 | .409 | .572 | .423 |
|  |  | D  (Depression) | Rho | **.384** | **.547** | **.394** | **-.313** | **.348** | **-.130** | **.526** |  |  |  |  | -.028 | .013 | .066 | -.022 |
|  |  |  | p | **.000** | **.000** | **.000** | **.000** | **.000** | **.001** | **.000** |  |  |  |  | .490 | .758 | .103 | .587 |
|  | **New onset** | A  (Somatization) | Rho | .022 | .007 | -.010 | -.041 | .022 | .015 | .035 | .087 | .023 | .019 | -.028 |  | **.525** | **-.311** | **.274** |
|  |  |  | p | .592 | .864 | .809 | .319 | .586 | .719 | .395 | .053 | .582 | .649 | .490 |  | **.000** | **.000** | **.000** |
|  |  | B  (Anxiety) | Rho | -.010 | -.058 | -.021 | .009 | -.003 | .068 | -.011 | .029 | .000 | -.034 | .013 |  |  | **-.443** | **.417** |
|  |  |  | p | .810 | .154 | .608 | .834 | .950 | .095 | .796 | .520 | .996 | .409 | .758 |  |  | **.000** | **.000** |
|  |  | C  (Social Disf.) | Rho | -.013 | -.005 | -.017 | -.017 | -.044 | -.035 | -.037 | -.026 | .015 | .023 | .066 |  |  |  | **-.422** |
|  |  |  | p | .752 | .901 | .676 | .680 | .277 | .393 | .367 | .561 | .718 | .572 | .103 |  |  |  | **.000** |
|  |  | D  (Depression) | Rho | .047 | -.007 | .011 | -.019 | .003 | .014 | .027 | .000 | -.047 | -.033 | -.022 |  |  |  |  |
|  |  |  | p | .251 | .873 | .780 | .648 | .934 | .732 | .506 | .993 | .247 | .423 | .587 |  |  |  |  |
